# Integrated transcriptome and *in vitro* analysis revealed anti-proliferative effect of citral in human stomach cancer through apoptosis

**DOI:** 10.1038/s41598-019-41406-8

**Published:** 2019-03-19

**Authors:** Sri Renukadevi Balusamy, Sivasubramanian Ramani, Sathishkumar Natarajan, Yeon Ju Kim, Haribalan Perumalsamy

**Affiliations:** 1 Department of Food Science and Biotechnology Sejong University, Gwangjin-gu, Seoul Republic of Korea; 2 Department of Horticulture, Sunchon National University, Suncheon, Republic of Korea; 3 Graduate School of Biotechnology, College of Life Science, Kyung Hee University, Yongin, 446- 701 Republic of Korea

## Abstract

Cancer is the second leading cause of death globally, particularly stomach cancer is third most common causes of cancer death worldwide. Citral possesses anti-tumor activity in various cancer cell lines, However its effect toward stomach cancer and its mechanism of action is have yet to be elucidated. The goal of the present study is to elucidate the role of citral in stomach cancer using transcriptome and *in vitro* approaches. We performed transcriptome analysis using RNA-seq and explored its capability to persuade apoptosis in AGS human stomach cancer cell lines *in vitro*. Furthermore, the enrichment and KEGG pathway results suggested that there are several genes involved to induce apoptosis pathway. Furthermore, our study also demonstrated that citral arrested colony formation and migration of cancer cells significantly than that of untreated cells. RNA-seq revealed a total of 125 million trimmed reads obtained from both control and citral treated groups respectively. A total number of 612 differentially expressed genes (DEGs) were identified which includes 216 genes up-regulated and 396 genes down-regulated genes after treatment. The enrichment analysis identified DEGs genes from transcriptome libraries including cell death, cell cycle, apoptosis and cell growth. The present study showed the significant inhibition effect upon citral by regulating various genes involved in signaling pathways, inhibits metastasis, colony formation and induced apoptosis both *in silico* and *in vitro*.

## Introduction

Stomach cancer is the third most among common cancer and second leading cause of death in the world^1,2^. The gastric cancer is often detected in an advanced stage and therefore the treatment was not highly successful and led to median overall survival^3^. A recent study was conducted to explore the epigenetic inhibitors of gastric cancer cell proliferation. Epigenetic modifications play a key role in gastric cancer proliferation which has been shown to link between development and progression of gastric cancer. When screening the library of epigenetic inhibitors, BET family of bromodomain was identified to show significant inhibitory effect of gastric cancer cells and could play a significant role in gastric cancer inhibition and clinical trials has also started recently. However, it has shown some resistant mechanisms in gastric cancer cells^4–7^. Chemopreventive technique is a promising approach which implies specific natural and chemical targets to suppress, prevent or reverse the precancerous cells from progressing into invasive cancer. There are certain dietary agents especially medicinal plants and phytoconstituents has been receiving interest as the possible chemopreventive agents over the past decades^8^.

Previous studies demonstrated the modulation of dietary habits with high intake of fruits, and vegetables rich in antioxidants can minimize the gastric cancer rate. The patients with gastric cancer are highly influenced by reactive oxygen species dependent lipid peroxidation that resulted due to insufficient antioxidants^9^. Specifically, fruits rich in vitamin c prevent the development of gastric cancer by delaying tumor progression in experimental animals^10^. These delaying actions may be due to any one of the combinational mechanisms: procarcinogen activation prevention, inactivating carcinogens, stimulating DNA repair mechanisms, down-regulating protooncogene expression, up-regulation of tumor suppressor genes, inhibition of cell proliferation, differentiation induction and promoting apoptosis, stimulation of immune system, and regulating transcription factors and abnormal signaling pathways^11,12^. Therefore, identifying the targets that can inhibit the growth of tumor cell by inducing apoptosis is one of the best strategies.

*Cymbopogan citratus* (D.C.) Stapf. commonly known as lemon grass oil distributed in all tropical and subtropical countries. Lemon grass is used for making soft drinks and as an aroma for making herbal tea. The aerial parts of lemon grass are also widely used in folk medicine to treat various diseases including digestive disorders, inflammation, diabetes, nervous disorders, and several other health problems^13^. It is also reported to possess antioxidants that scavenge free radicals and can be used in the prevention of several life-threatening diseases such as atherosclerosis, heart diseases, cancer and arthritis in which reactive oxygen species (ROS) play a crucial role. ROS generation is also associated with diseases in gastrointestinal tract^13,14^. Next generation sequencing (NGS) made feasible to analyze large-scale screening transcriptome that discovered genes at genome level without the reference genome sequences. Transcriptome analysis along with bioinformatic data mining tools provides the platform to simultaneously interpret various genes, identify the targets and its interactions after treatments. There are several previous studies demonstrated the role of citral on inhibiting the growth of cancer cells by targeting molecular signaling pathways including small-cell lung cancer; breast cancer-MDA-MB-231; Colorectal cancer^15,16^. This is the first report to combine both RNA seq and *in vitro* analysis to prove the effect of citral on suppressing stomach cancer growth by promoting apoptosis.

## Results and Discussion

### Isolation of citral from *Cymbopogan* *citratus* oil

The major active constituent citral was isolated identified through various spectroscopic analyses; including electron ionized mass spectrometry (EI-MS) and nuclear magnetic resonance (NMR) spectroscopy. Citral was identified based on the following evidence: a colorless oil; UV (methanol): λ_max_ nm = 233; EI-MS (70 eV), *m*/*z* (% relative intensity): 152 [M]^+^ (6.4), 137 (3.8), 94 (12.5), 84 (24.6), 69 (100), 59 (3.40), 41 (87.3), ^1^H NMR (DMSO, 600 MHz): δ 1.62 (3 H, s), 1.67 (3 H, d, *J* = 1.2 Hz), 2.00 (2 H, s), 2.18 (3 H, m), 2.62 (2 H, t), 5.20 (1 H, m), 5.83 (1 H, t), 9.96 (1 H, d, *J* = 8.16 Hz). ^13^C NMR (CDCl_3_, 150 MHz): δ 192.4 d (C-1), 166.1 s (C-3), 134.4 s (C-7), 128.4 d (C-2), 123.5 d (C-6), 40.4 t (C-4), 26.9 t (C-5), 25.1 q (C-8), 18.1 q (C-9), 17.8 q (C-10) (Supplementary Fig. S1).

### Citral inhibited cancer viability by altering cell morphology

**Figure 1:**
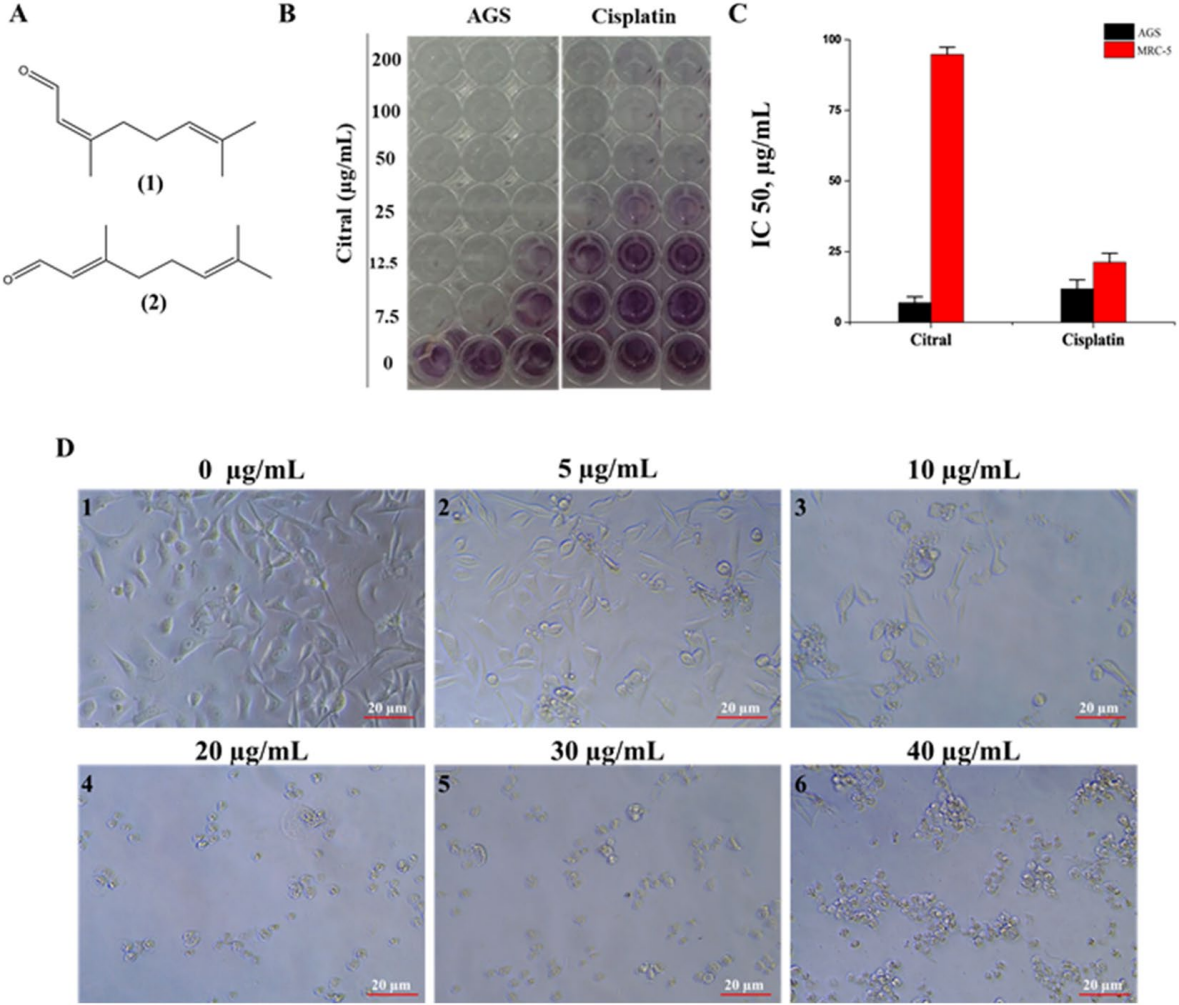
Decreased cell viability and morphological changes after citral treatment. (**A**) The chemical structure of cis- (neral) and trans-citral (geranial). (**B**) The image was captured right after performing MTT assay. (**C**) Inhibitory concentration (IC_50_) of citral towards AGS cancer cells was compared with positive control cisplatin. X-axis indicate compounds treated for cell lines and Y-axis indicate IC_50_. Cell toxicity of citral was compared with normal cell line MRC-5. (**D**) Morphological characteristics of AGS cell lines were observed after the treatment.

The inhibition of cell proliferation occurred after treatment with citral (Fig. 1A) at various concentrations (7.5, 12.5, 25, 50, 100, 200 µg/mL) in AGS cell lines. The photographs and calculation of IC_50_ were performed after 48 h of treatment (Fig. 1B–D). The 5 µg/mL of citral treated cells showed gradual change in the morphology and extended its determined alteration at 40 µg/mL respectively. All the treated samples tend to show distinct phenotype including shrunken cells, shapeless and significantly reduce in cell number. Whereas, non-treated cells showed no difference in morphology and displayed 90% confluent cells. Various plant essential oils decrease the various cancer cell growth by inducing decrease in cell number, cell morphology and cytoplasmic vacuolation and nuclei^17,18^.

### RNA sequencing analysis

To identify the expression pattern of apoptosis related genes after citral treatment in AGS cell line RNA sequencing were performed. At first, isolated RNA quality and concentration was measured by using Agilent 2100 BioAnalyzer. The high-quality samples were considered to perform paired end (PE) RNA sequencing with control (non-treated) and citral treated samples using the Illumina Hiseq 2500 platform. Supplementary Fig. 2 represents quality of RNA isolation and Phred score results of the control and citral (20 µg/mL) in AGS. After sequencing, the quality of reads of each sample were evaluated using FastQC (version 1.0.0) based on Phred quality score (ref: https://www.ncbi.nlm.nih.gov/pubmed/9521921). A total of 140 million paired end reads with average of 70 million reads per sample were generated from RNA sequencing and their summary of transcriptome statistics were described in Table 1. Furthermore, low quality reads and adaptor sequences were removed with high stringency filtering yielded that 125 million reads (average of 63 million per accession). In total, 85.57% and 80.49% reads was mapped (average of 83%) on the reference h19 genome to the control and citral treated respectively. The raw reads in FASTQ format which supporting this study obtained from paired-end (PE) RNASeq libraries have been deposited into Sequence Read Archive (SRA) database under National Center for Biotechnology Information (NCBI) accession number SRP150561 (https://www.ncbi.nlm.nih.gov/sra/SRP150561).

**Table 1:** The statistics summary of RNA-Seq libraries.

Library	Total reads	Reads length (bp)	Trimmed reads	Mapped reads	Mapping reads (%)
Control	75, 171,336	101	74,990,756	64,170,781	85.57
Treated	65,905,230	101	51,385,566	41,357,663	80.49

### Differential expression analysis

The cufflinks program was used to normalize and measure relative abundance of reads through fragments per kilobase million (FPKM) and density expression levels of control and citral treated libraries based on log_10_ value of FPKM were represented in Supplementary Fig. 3. Then, while comparing normal with citral treated AGS cells, a total of 612 differentially expressed genes (DEGs) were identified based on statistical significance values of fold change ≥2 and p-value < 0.05. Among all those genes, 216 and 396 genes were up-regulated and down-regulated respectively. The significantly expressed genes were shown as Supplementary Fig. 3 and entire list of identifying differently expressed genes were given as Supplementary File S6.

### Enrichment analysis

**Figure 2:**
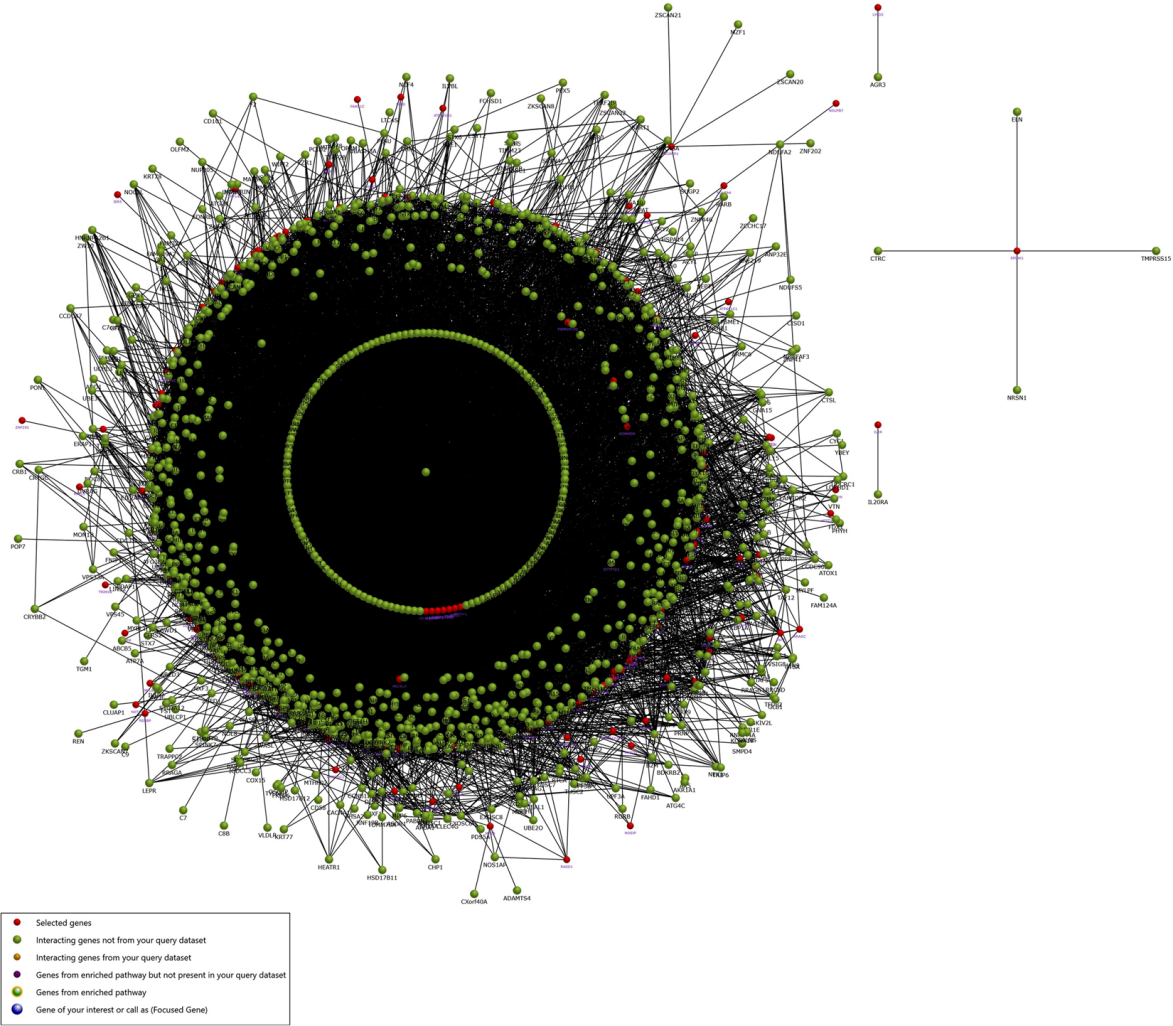
Functional enrichment based interaction of up-regulated DEGs with related external genes.

**Figure 3:**
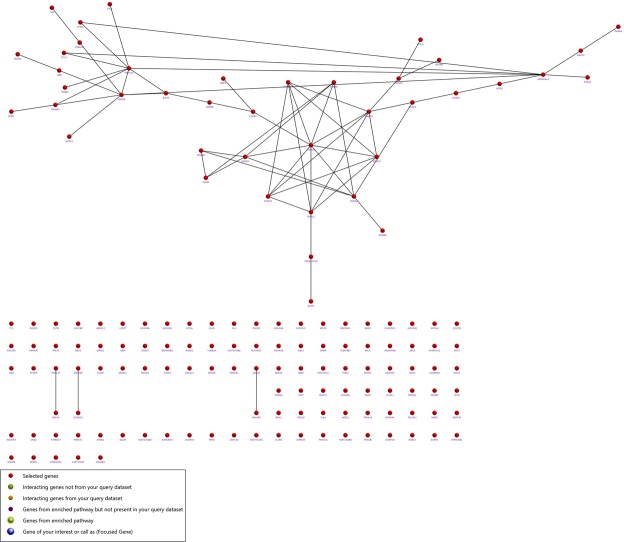
Functional enrichment based interaction network of DEGs of citral treatment groups.

**Figure 4:**
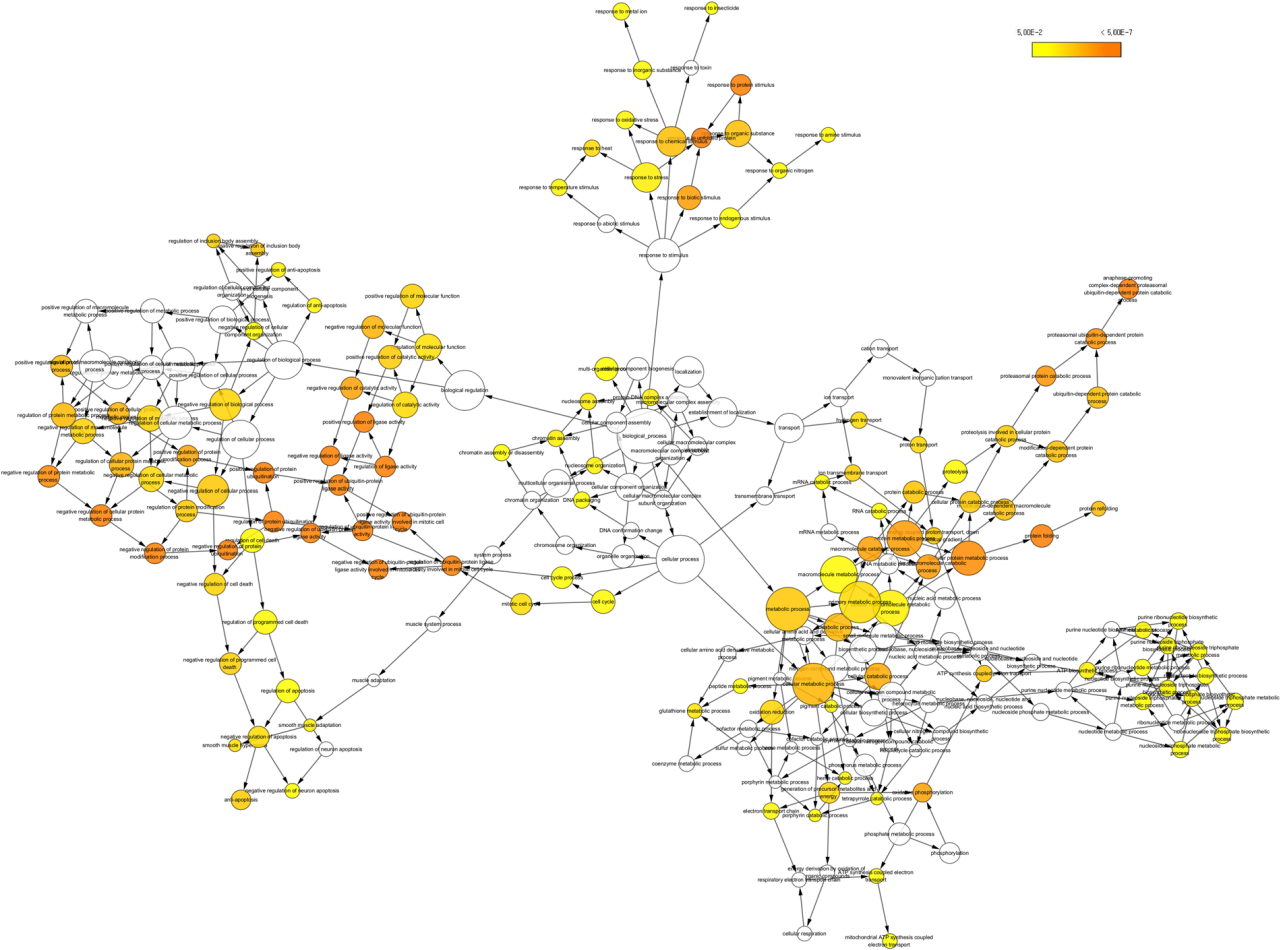
Network visualization of significantly overrepresented GO terms.

**Figure 5:**
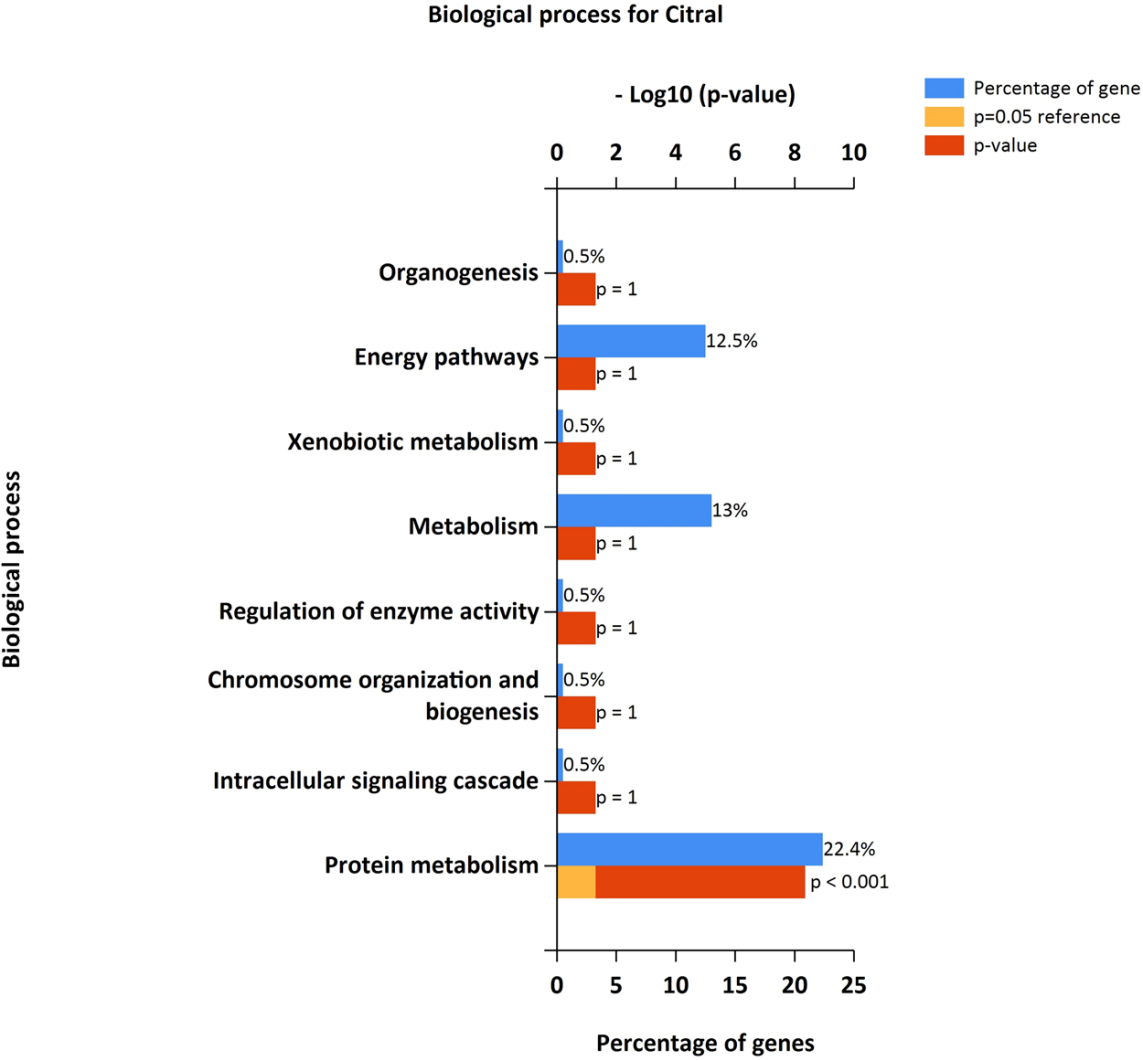
Functional gene enrichment analysis of identified DEG as biological process.

**Figure 6:**
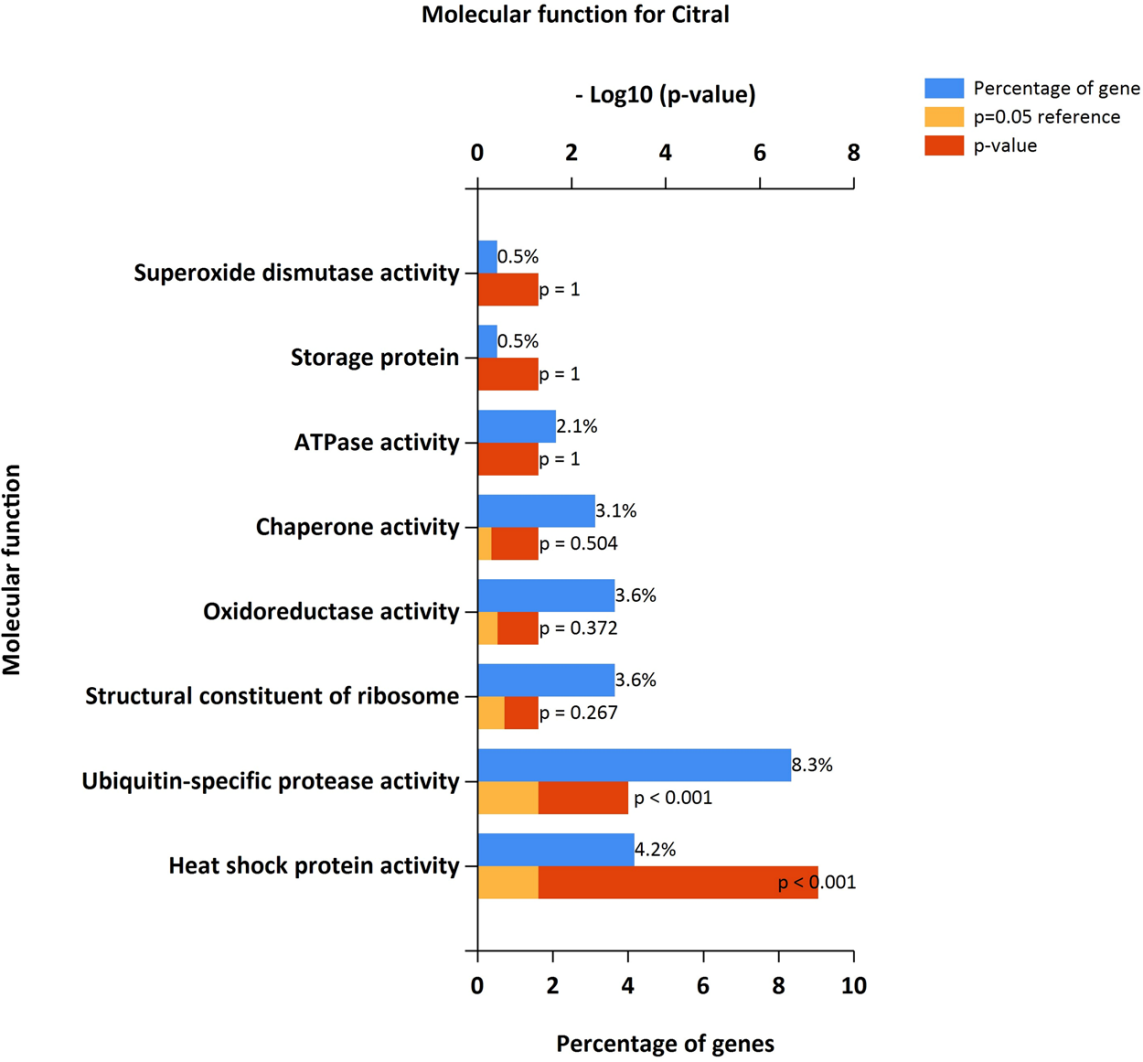
Functional gene enrichment of DEG as molecular function.

**Figure 7:**
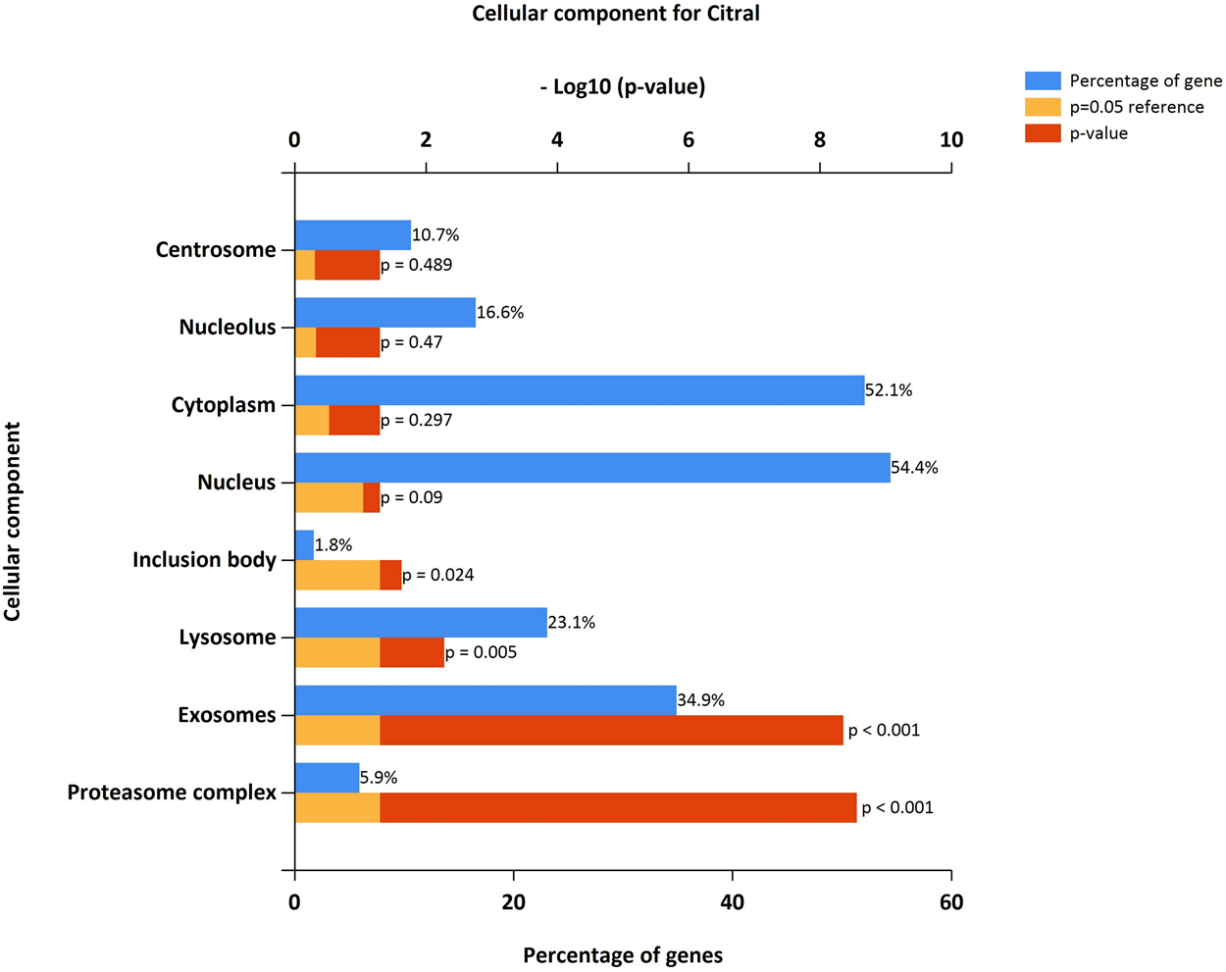
Functional gene enrichment of DEG as cellular component.

The identified DEGs were investigated into functional enrichment analysis. Network of DEG and network between up-regulated DEG and relative external genes is shown (Figs 2 and 3; Supplementary Files 1 and 2) based on gene enrichment. The GO terms mainly include three major categories of biological process (BP), molecular function (MF) and cellular component (CC).The gene ontology of 216 DEG resulted in 196 recognized genes thus further analysis is proceeded with these 196 genes with GO terms (Fig. 4). The detail information is given in the Supplementary Files 3–5. The enrichment results are projected here for BP (Fig. 5), MF (Fig. 6) and CC (Fig. 7) respectively. Further analysis of GO subcategories, observed that *PCK2*, *IFI6*, *LGALS3BP* and *EDAR* genes were mainly involved in down-regulation upon citral treatment while *PLCG2*, *ZFAND2* and *HSPB1* DEGs genes were up-regulated (Supplementary File S6). Furthermore, KEGG pathway analysis revealed that significantly enriched DEGs were grouped into 46 known pathways (Supplementary File S7).

### Enrichment analysis of DEGs with associated apoptosis

Cell death specifically apoptosis is one of the most vital studied candidates among cell biologists. Therefore, detail study of pathways involved in cell death is essential to gain insights into pathogenicity of the disease and can pave the way to identify the possibilities of treating the disease. KEGG analysis revealed that MAPK signaling pathway, NF-kappa B signaling pathway, p53 signaling pathway, PI3-K-Akt signaling pathway, pathways in cancer, and prostate cancer pathways are mostly enriched in up and down-regulated genes which identified between normal and citral treated samples in AGS. Among them, cell cycle, pathways in cancer, and MAPK signaling pathways were observed as mostly enriched pathways. In addition, several common genes were identified in these pathways were listed in Table 2. Among them, cell cycle (hsa04110) and cancer (hsa05200) occupied several down-regulated DEG genes. The genes involved in cell cycle synthesis are very crucial for the inhibition of cancer cell progression^19^. For example, cyclin-dependent kinases (CKD2) are the most important regulators for the transition and cell cycle progression and play a significant role in regulating cell cycle division including centromere duplication, DNA synthesis, G1-S transition, and modulation of G2 progression^20–22^. Furthermore, the enrichment analysis and KEGG pathway results indicated that, there are several genes involved to initiate apoptosis pathway that are directly/indirectly related to the treatment of citral. In addition, enrichment analysis identified DEGs genes from transcriptome libraries were mainly involved in cell death, cell cycle, apoptotic process, and cell growth (Table 3).

**Table 2:** KEGG enrichment analysis of identified DEG genes from control and citral treated samples in AGS.

Differential expressed genes	KEGG	Pathway	Genes involved	Log2(fold change)
**Up-regulated genes**	hsa04101	**MAPK signaling pathway**	**HSPA1A**, **HSPA1B**, **GADDA45B**	8.09
	hsa04064	**NF-kappa B signaling pathway**	**PLCG2**	5.42
	hsa05200	**Pathways in cancer**	**PLCG2**, **HSP90AA1**, **CKS2**	5.42
	hsa05215	**Prostate cancer**	**HSP90AA1**	3.57
	hsa04151	**PI3K-Akt signaling pathway**	**HSP90AA1**, **ILR6**	2.54
	hsa04115	**p53 signaling pathway**	**GADD45B**	2.54
	hsa04210	**Apoptosis**	**TNFRSF10D**	1.85
	hsa03040	**Spliceosome**	**SYF2**	1.67
**Down-regulated genes**	hsa05200	**Pathways in cancer**	**MSH6**, **MSH2**, **COL4A5**, **MMP1**, **CDK2**, **CCNE2**, **LAMB1**, **RAD51**, **PDGFRA**	−1.17
	hsa04110	**Cell cycle**	**CCNE1**, **CCNB3**, **CHEK1**, **CCNB1**, **CDK2**, **CDC7**, **E2F1**, **WEE1**, **CCNA2**, **MCM3**, **TTK**, **MCM6**, **CDC6**, **PKMYT1**, **BUB1B**, **PLK1**, **ESPL1**	−1.25
	hsa05222	**Small cell lung cancer**	**CCNE1**, **LAMB1**, **E2F1**	−1.25
	hsa04151	**PI3K-Akt signaling pathway**	**CCNE1**, **COL4A5**, **COL27A1**, **LAMB1**, **PDGFRA**, **PCK2**	−1.25
	hsa04115	**p53 signaling pathway**	**CCNB3**, **CCNB1**, **CDK2**, **CCNE2**, **RRM2**, **SERPINE1**	−1.27
	hsa05210	**Colorectal cancer**	**MSH2**	−1.65
	hsa05202	**Transcriptional mis-regulation in cancer**	**WHSC1**, **NUPR1**	−1.68
	hsa04010	**MAPK signaling pathway**	**CACNG8**, **MAP3K5**, **DUSP6**, **RPS6KA2**, **RPS6KA5**, **PDGFRA**	−1.76
	hsa05219	**Bladder cancer**	**MMP1**, **VEGFA**, **E2F1**	−1.80
	hsa03320	**PPAR signaling pathway**	**MMP1**, **SLC27A2**, **PCK2**	−1.80
	hsa01212	**Fatty acid metabolism**	**HADH**, **E2F2**	−1.93
	hsa05205	**Proteoglycans in cancer**	**VEGFA**, **VTN**	−1.97
	hsa04064	**NF-kappa B signaling pathway**	**TRAF5**	−2.15
	hsa05215	**Prostate cancer**	**E2F1**	−2.24
	hsa04152	**AMPK signaling pathway**	**CCNA2**, **CFTR**, **PCK2**	−2.26
	Hsa05223	**Non-small cell lung cancer**	**E2F2**	−4.03

**Table 3:** Functional enrichment analysis of DEGs genes involved in apoptosis pathway after citral treatment.

DEG							
Go terms	Category	Number of genes	Go id	p-value	Up-regulated genes	p-value	Down- regulated genes
**Response to unfolded protein**	**BP**	8	GO:0006986	1.79E-09	**HSPA4L**, **DNAJA1**, **HSPA8**, **HSPH1**, **HSP90AA1**, **DNAJB1**, **DNAJB4**, **HSPB1**	NA	NA
**Negative regulation of programmed cell death**	**BP**	2	GO:0043069	0.62	**PLCG2**	0.33	**KANK2**
**Negative regulation of cell death**	**BP**	4	GO:0060548	0.77	**HSPA1A**, **CACYBP**, **HSPA1B**	0.57	**SRGAP2**
**Regulation of cell cycle**	**BP**	19	GO:0051726	1.64E-05	**GADD45B**, **HSPA8**	1.64E-05	**E2F2**, **RBL1**, **MYBL2**, **PLK1**, **PLK1**, **GTSE1**, **WEE1**, **MX2**, **PKMYT1**, **CCNF**, **CDC25A**, **CCNB2**, **PKD2**, **MASTL**, **SRC**, **NUPR1**, **RPS6KA2**
**Negative regulation of programmed cell death**	**BP**	2	GO:0043069	0.62	**PLCG2**	0.33	**KANK2**
**Positive regulation of cell death**	**BP**	1	GO:0010942	0.12	**PRODH**	NA	NA
**Negative regulation of cell death**	**BP**	4	GO:0060548	0.77	**HSPA1A**, **CACYBP**, **HSPA1B**	0.57	**SRGAP2**
**Intracellular membrane-bounded organelle**	**CC**	10	GO:0043231	NA	NA	0.67	**MCM3**, **CHAF1A**, **SAMD9**, **OAS2**, **SLC7A5**, **DBF4B**, **DNMT3B**, **LIG1**, **BARD1**, **ATP8A1**
**Negative regulation of cell growth**	**BP**	2	GO:0030308	NA	NA	0.87	**APBB2**, **CAPRIN2**
**Negative regulation of catalytic activity**	**BP**	1	GO:0043086	0.64	**CLN3**	NA	NA
**Cell cycle arrest**	**BP**	4	GO:0007050	NA	NA	0.64	**APBB2, PKD2, PKD1, BARD1**
**Negative regulation of growth**	**BP**	2	GO:0045926	0.40	**MT1X**, **MT2A**	NA	NA
**Cellular protein metabolic process**	**BP**	5	GO:0044267	NA	NA	0.64	**IGFBP4**, **HSPG2**, **IGFBP2**, **UBE2L6**, **LYZ**
**Positive regulation of cellular protein metabolic process**	**BP**	1	GO:0032270	NA	NA	0.76	**UHRF1**

BP: Biological process; CC: Cellular component; NA Not available.

### Regulation of apoptosis related genes under citral treatment

RNA-seq data after citral treatment resulted in involvement of both up- and down- regulation of apoptosis pathway related genes. There are several genes up-regulated upon citral treatment and are involved in biological function (Table 4). Our results were also in agreement with several previous studies in various cancer types. For instance, IL-24 function is to induce cancer cell apoptosis through the down-regulation of Bcl-2 expression which is a key factor involved in mitochondrial mediated apoptosis^23^. Similarly, the intron region of p53 directly binds to the ribosomal protein RPS27L and induced apoptosis in various cancer models both *in vitro* and *in vivo*^24^. Apoptosis mechanism is a normal programmed death process which is very crucial for maintaining disease free body. Several genes that are associated with negative regulation of apoptosis can inhibit the apoptosis process and thus result in the cancer cell progression. Our result suggests that upon citral treatment, several genes involved in negative regulation of apoptosis pathways including *IF16*, *DHRS2*, *IFIT3*, *CEACAM5*, *PLK*, *1CDK1* and so on. (Table 5) are down-regulated upon citral treatment. Apart from this, there is down-regulation of genes involved in positive regulation; indicating that these genes have less significant role in inducing apoptosis after citral treatment.

**Table 4:** The identified up-regulated genes list after citral treatment in AGS cell lines.

Go terms	Go ids	Category	p-value	Genes
**BP**	GO:0006915	**Apoptotic process**	0.54	**IL24**
**BP**	GO:0043066	**Negative regulation of apoptotic process**	0.46	**CLN3**
**BP**	GO:0043066	**Negative regulation of apoptotic process**	0.46	**UBA52**
**BP**	GO:0043065	**Positive regulation of apoptotic process**	0.52	**UBA52**
**MF**	GO:0008656	**Cysteine-type endopeptidase activator activity involved in apoptotic process**	0.36	**RPS27L**
**BP**	GO:0006919	**Activation of cysteine-type endopeptidase activity involved in apoptotic process**	0.73	**RPS27L**
**BP**	GO:0043065	**Positive regulation of apoptotic process**	0.52	**SOD1**
**BP**	GO:0043066	**Negative regulation of apoptotic process**	0.46	**SERPINB2**
**BP**	GO:0043065	**Positive regulation of apoptotic process**	0.52	**HMOX1**
**BP**	GO:0043066	**Negative regulation of apoptotic process**	0.46	**UBB**
**BP**	GO:0043065	**Positive regulation of apoptotic process**	0.52	**UBB**
**BP**	GO:0043065	**Positive regulation of apoptotic process**	0.52	**GADD45B**
**BP**	GO:0006915	**Apoptotic process**	0.54	**GADD45B**
**BP**	GO:0043066	**Negative regulation of apoptotic process**	0.46	**DNAJA1**
**BP**	GO:0043065	**Positive regulation of apoptotic process**	0.52	**DNAJA1**
**BP**	GO:0043065	**Positive regulation of apoptotic process**	0.52	**CLU**
**BP**	GO:0042981	**Regulation of apoptotic process**	0.59	**DEDD2**
**BP**	GO:0043065	**Positive regulation of apoptotic process**	0.52	**OSGIN1**
**BP**	GO:0010664	**Negative regulation of striated muscle cell apoptotic process**	0.26	**BAG3**
**BP**	GO:0043066	**Negative regulation of apoptotic process**	0.46	**BAG3**
**BP**	GO:0043066	**Negative regulation of apoptotic process**	0.46	**HSPB1**

BP: Biological process; MF: Molecular function.

**Table 5:** The identified down-regulated genes list after citral treatment in AGS cell lines.

Go terms	Go ids	Category	p-value	Genes
**BP**	GO:0006915	**Apoptotic process**	0.45	**EDAR**
**BP**	GO:0043154	**Negative regulation of cysteine-type endopeptidase activity involved in apoptotic process**	0.95	**IFI6**
**BP**	GO:0043065	**Positive regulation of apoptotic process**	0.33	**IFIT2**
**BP**	GO:0043066	**Negative regulation of apoptotic process**	0.50	**DHRS2**
**BP**	GO:1990086	**Lens fiber cell apoptotic process**	0.04	**E2F2**
**BP**	GO:0043066	**Negative regulation of apoptotic process**	0.50	**IFIT3**
**BP**	GO:0043066	**Negative regulation of apoptotic process**	0.50	**CEACAM5**
**BP**	GO:0043524	**Negative regulation of neuron apoptotic process**	0.09	**XRCC2**
**BP**	GO:0043066	**Negative regulation of apoptotic process**	0.50	**PLK1**
**BP**	GO:0043065	**Positive regulation of apoptotic process**	0.33	**TOP2A**
**BP**	GO:0006915	**Apoptotic process**	0.45	**CDK1**
**BP**	GO:0043066	**Negative regulation of apoptotic process**	0.50	**CDK1**
**BP**	GO:0006919	**Activation of cysteine-type endopeptidase activity involved in apoptotic process**	0.88	**IFI27**
**BP**	GO:0006915	**Apoptotic process**	0.45	**ESPL1**
**BP**	GO:0043154	**Negative regulation of cysteine-type endopeptidase activity involved in apoptotic process**	0.95	**LAMP3**
**BP**	GO:0043066	**Negative regulation of apoptotic process**	0.50	**ASNS**
**BP**	GO:0006915	**Apoptotic process**	0.45	**BUB1B**
**BP**	GO:0006915	**Apoptotic process**	0.45	**PLSCR1**
**BP**	GO:0042981	**Regulation of apoptotic process**	0.67	**INHBE**
**BP**	GO:0006915	**Apoptotic process**	0.45	**TPX2**
**BP**	GO:0006919	**Activation of cysteine-type endopeptidase activity involved in apoptotic process**	0.88	**TNFSF15**
**BP**	GO:0006915	**Apoptotic process**	0.45	**BUB1**
**BP**	GO:0043065	**Positive regulation of apoptotic process**	0.33	**APBB2**
**BP**	GO:0043066	**Negative regulation of apoptotic process**	0.50	**APBB2**
**BP**	GO:0006915	**Apoptotic process**	0.45	**MCM2**
**BP**	GO:0006915	**Apoptotic process**	0.45	**NTN1**
**BP**	GO:0006915	**Apoptotic process**	0.45	**TNS4**
**BP**	GO:0002903	**Negative regulation of B cell apoptotic process**	0.53	**AURKB**
**BP**	GO:0043066	**Negative regulation of apoptotic process**	0.50	**MAD2L1**
**BP**	GO:0043065	**Positive regulation of apoptotic process**	0.33	**PLEKHG2**
**BP**	GO:0006915	**Apoptotic process**	0.45	**TRAF5**
**BP**	GO:0042981	**Regulation of apoptotic process**	0.67	**TRAF5**
**BP**	GO:0043065	**Positive regulation of apoptotic process**	0.33	**MELK**
**BP**	GO:0006915	**Apoptotic process**	0.45	**MELK**
**BP**	GO:0006915	**Apoptotic process**	0.45	**CHI3L1**
**BP**	GO:0043065	**Positive regulation of apoptotic process**	0.33	**SRC**
**BP**	GO:0043066	**Negative regulation of apoptotic process**	0.50	**SRC**
**BP**	GO:0043154	**Negative regulation of cysteine-type endopeptidase activity involved in apoptotic process**	0.95	**SRC**
**BP**	GO:0042981	**Regulation of apoptotic process**	0.67	**TNFRSF1B**
**BP**	GO:0006915	**Apoptotic process**	0.45	**KANK2**
**BP**	GO:0043066	**Negative regulation of apoptotic process**	0.50	**BTC**
**BP**	GO:0043065	**Positive regulation of apoptotic process**	0.33	**BARD1**
**BP**	GO:0043066	**Negative regulation of apoptotic process**	0.50	**BARD1**
**BP**	GO:2000352	**Negative regulation of endothelial cell apoptotic process**	0.10	**SERPINE1**
**BP**	GO:0043524	**Negative regulation of neuron apoptotic process**	0.09	**TERT**
**BP**	GO:2000352	**Negative regulation of endothelial cell apoptotic process**	0.10	**TERT**
**BP**	GO:0043065	**Positive regulation of apoptotic process**	0.33	**NUPR1**
**BP**	GO:0043065	**Positive regulation of apoptotic process**	0.33	**NTSR1**
**BP**	GO:0043066	**Negative regulation of apoptotic process**	0.50	**NTSR1**
**BP**	GO:0043065	**Positive regulation of apoptotic process**	0.33	**RPS6KA2**
**BP**	GO:0043065	**Positive regulation of apoptotic process**	0.33	**TGM2**
**BP**	GO:0043066	**Negative regulation of apoptotic process**	0.50	**TGM2**

BP: Biological process.

### Citral reduced stomach cancer cell proliferation by inhibiting colony formation

**Figure 8:**
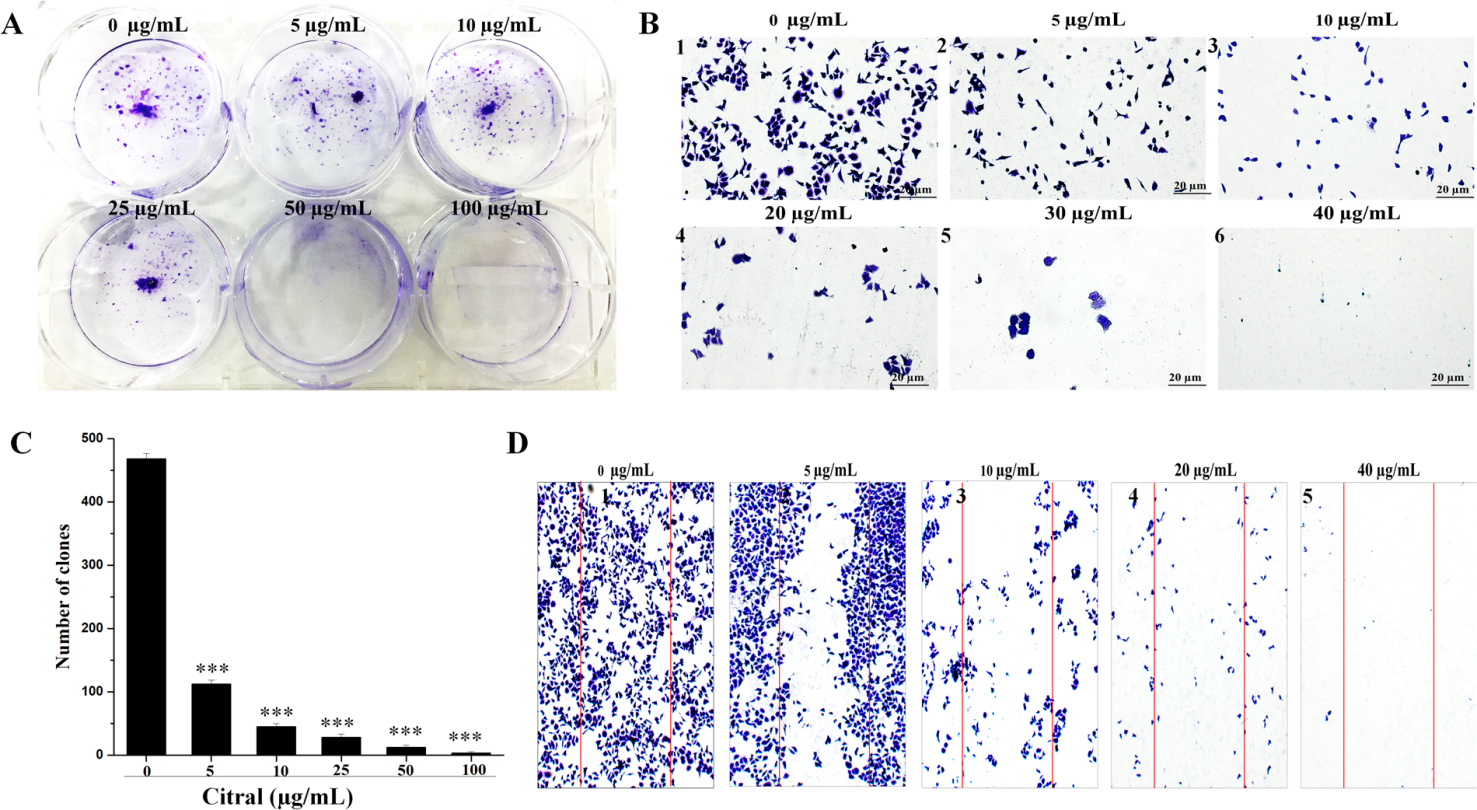
The colony progress and invasive ability of AGS citral treatment (with or without) (**A**,**B**). Cells were seeded uniformly (1 × 10^3^) and grown for 4 days. The crystal violet staining were used to detect the colonies. Based on dose-dependent (citral) the cells clearly exhibited growth inhibition. (**C**) The total number of colonies in with or without treatment were measured using imageJ software and plotted graphically (Fig. 2C). (**D**) The effect of citral has reduced down the migration cells associated to that of untreated cells and migration ability decreased with increasing concentration of citral treatment (Fig. 2D2–D5). The images are representative of three independent replicates. Each bar represents the mean ± SE of duplicate samples of three independent experiments (***P < 0.001using student’s t-test).

Colony formation assay is performed to identify the citral ability to inhibit the growth of AGS cells as well as to indirectly monitor the cell death^25^. For the treatment of the cell lines, different concentration of citral from 5, 10, 25, 50, 100 µg/mL were used respectively. The experiment was carried out for continuously for 7 days in a 6-well plate by changing fresh media and every 2–3 days. The non- treated cells showed colony formation and amplified the cell number with increased colony formation (Fig. 8A,B). Whereas, the treated cells even at 5 µg/mL showed decreased in colony number and induced the cell death thus specifying that citral has a role to be used as anti-proliferative drug. The total number of colonies were calculated using cell counter (imageJ software) and it was plotted (Fig. 8C).

### Migration ability of cancer cell was inhibited by citral treatment

We have assessed the invasion and metastatic stage of AGS cell line after citral treatment. The growth effect and migration was affected in cancer cell by the treatment of citral in various concentration (0, 5, 10, 20, 30, 40 µg/mL) (Fig. 8D2–D5). In case of control, cancer cells exposed completely grown migrated cells (Fig. 8D1). The cancer cell progression is the sequence of occurrences essential to the development of metastasis and cell invasion. Once the cells can invade the nearby tissues, these cells pass through the base membrane and extracellular matrix and further penetrate to the lymphatic and vascular circulation. Ultimately, cells grow in the new area and proliferate to produce secondary tumor^26^. However, citral treatment inhibited the cell invasion and migration of AGS cells and thus prevented these consequences.

### Citral induced apoptotic effect in human stomach cancer cell lines *in vitro*

**Figure 9:**
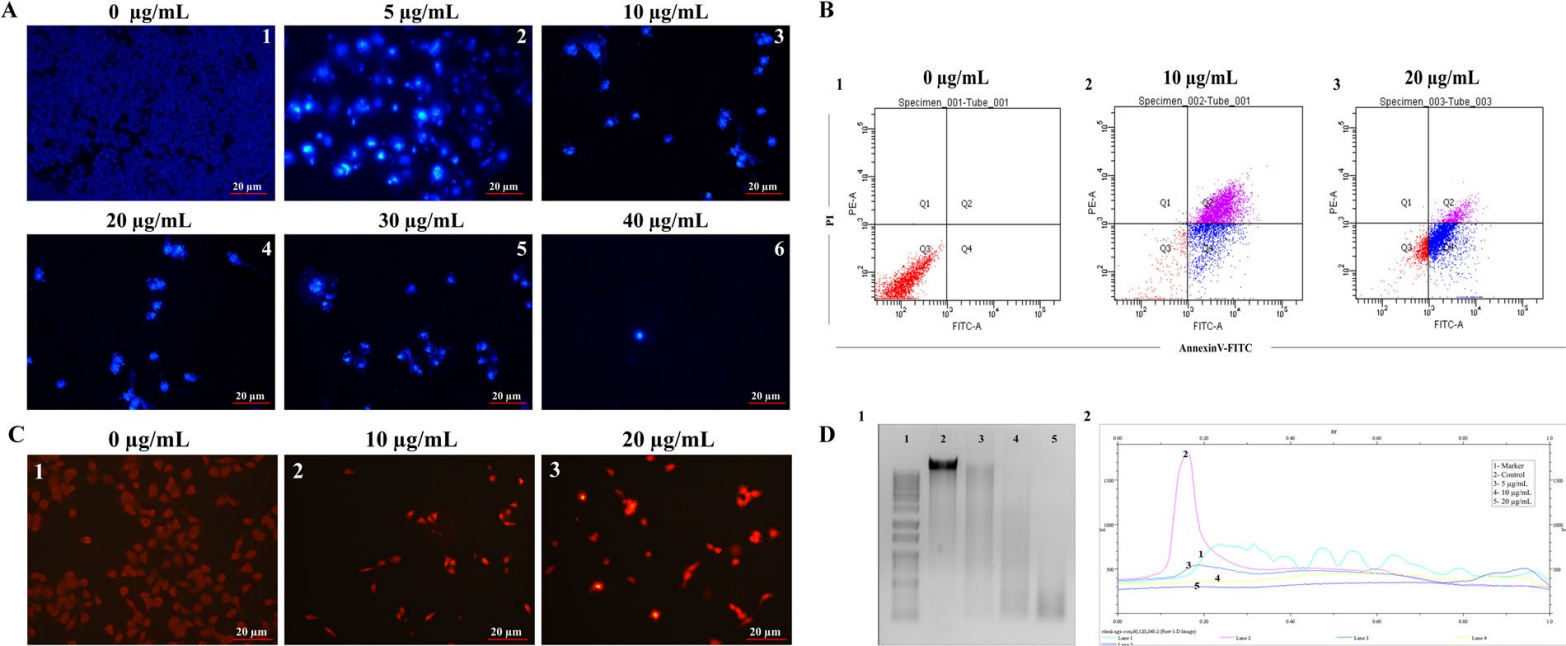
Citral induced apoptosis in AGS cell lines. (**A**) DNA condensation and nuclear staining of AGS cells with or without citral treatment. (**B**) Flow cytometry analysis of cancer cells with or without treatment. Expressive figures displaying the population of live cells (annexin V− PI−), early apoptotic (annexin V+ PI−), late apoptotic cells (annexin V+ PI+) and necrotic cells (annexin V− PI+). (**C**) PI staining of AGS cells with or without citral treatment. The cells remained intact don’t allow cells to stain with the dye. However, the damage cells were stained with PI and indicated the apoptotic cells. (**D**) DNA fragmentation analysis was carried out after isolation of genomic DNA from AGS with or without different concentration of citral treatment. 1. DNA ladder 1 kb; 2. Control; 3. 5 µg/mL; 4. 10 µg/mL; 5. 20 µg/mL.

The human body generates 10–100 billion cells every day and the equal number of cells dies to maintain homeostasis. Under this condition, cell death occurs mainly by undergoing apoptosis which is silent process^27^. However, cancer cells modulate the apoptosis process by transcriptionally, translationally and even by post-translational modification and thus resulting in cancer cells to escape apoptotic process^28^. Therefore, identifying the target that increase the apoptosis in cancer cells is one of the best strategies^29,30^. Our RNA-seq data also demonstrated there is occurrence of apoptosis after citral treatment (Supplementary File S6). In order to confirm it *in vitro*; we performed various assays that can confirm the apoptosis. The hoechst staining was performed to study the condensation and apoptosis happened after treatment of citral to cancer cells. Our results demonstrated that upon citral treatment, there is visible chromatin condensation and degradation of nuclei of AGS cells happened at different concentration of citral treatment. In untreated state, the cell membrane present in its undamaged form and the dye is unable to diffuse into the nuclei and has no fluorescence (Fig. 9A1). However in 5 µg/mL of citral treatment, cells started to show chromatin condensation and fragmented/shrunken nuclei like structures due to damage of cell membrane. The intensity of damage increase when the concentration of citral increases (Fig. 9A2–A6). When looking inside the cell, one of the most obvious features of apoptosis is condensation of the nucleus and its fragmented segments^31^. Annexin-V-FITC results also exhibited that citral inhibited together early and late apoptosis in AGS cells. In the concentration of 10 µg/mL citral showed maximum accumulation of late apoptosis (Q2) and moderate number of cells undergone early apoptosis (Q4) (Fig. 9B2). Whereas, 20 µg/mL of citral treated cells exhibited maximum number of early apoptotic cells (Q2) and moderate amount of late apoptosis respectively (Q4) (Fig. 9B3). Later, the propidium iodide (PI) staining was performed to determine the dead cells and live cells. This dye is particularly permeable in the dead cells because the dye can easily penetrate the damaged cell and thus resulted in the bright fluorescence. The cells treated with citral (10 and 20 µg/mL) showed bright fluorescence with decrease in cell number and reduced cell viability (Fig. 9C2–3). In contrast, the cells stained without treatment showed the absence of fluorescence clearly indicating that cells are intact (Fig. 9C1). Finally, the DNA fragmentation after citral treatment was assessed. The untreated cells showed the presence of intact DNA however, the cells treated with different concentration (5, 10 and 20 µg/mL) showed there is gradual increase in degradation of DNA occurred (Fig. 9D). The breakdown of DNA into small fragments is one of the characteristic features of apoptosis^31,32^.

## Materials and Methods

### Reagents, chemicals and cell lines

Commercially available anticancer drug cisplatin, and MTT (Thiazolyl blue tetrazolium bromide) were obtained from Sigma-Aldrich (St. Louis, MO). The culture medium, serum and phosphate buffer were purchased from by Life Technologies (Grand Island, NY). Antibiotic-antimycotic solution and 0.5% trypsin-ethylenediaminetetraacetic acid (EDTA) was obtained from Invitrogen (Grand Island, NY, USA).Citral essential oil was acquired from Berjé (Carteret, NJ, USA). All the other chemicals and reagents used in this study were of reagent-grade quality and available commercially. The cancer cell lines (AGS: ATCC-CRL-1739), Human lung normal cell lines (MRC-5: ATCC-CCL-171) were cultured in RPMI 1640 containing 10% FBS and 1% antibiotic-antimycotic solution under 5% CO2 and 95% air at 37 °C, whereas MRC-5 cell lines were cultured with DMEM containing 10% FBS, 1% antibiotic-antimycotic solution and 1% glutamine.

### Medium pressure liquid chromatography (MPLC)

The lemongrass oil (5 g) was monitored by TLC on silica gel plates (Silica gel 60 F_254_) developed with hexane and ethyl acetate solvent system. Based on TLC pattern result it was separated by MPLC using a BiotageIsolera apparatus equipped with a UV detector at 254 nm and 365 nm and a column cartridge SNAP (100 g silica gel) with column volume 132 mL. Separation was achieved with a gradient of hexane and ethyl acetate [100:0 (264 mL), 9:1 (396 mL), 8:2 (396 mL), 7:3 (660 mL), 6:4 (264 mL), 5:5 (264 mL), 3:7 (264 mL), and 1:9 (132 mL) by volume] at a flow rate 25 mL/min to provide 105 fractions (each about 22 mL). Column fractions were monitored by TLC on silica gel plates (Silica gel 60 F_254_) developed with hexane and ethyl acetate (8:2 by volume) mobile phase. Fractions with similar *R*_f_ values on the TLC plates were pooled. Spots were detected by spraying with 5% sulfuric acid and then heating on a hot plates stated previously. A preparative high-performance liquid chromatography (HPLC) was performed to separate the constituents from active fractions. The column was a 7.8 mm i.d. × 300 mm µBondapak C18 (Waters, Milford, MA, USA) with a mobile phase of methanol and water (85:15 by volume) at a flow rate of 1 mL min^−1^. Chromatographic separation was monitored using a UV detector at 233 nm. Finally, an active constituent 1(citral) (28 mg) was isolated at a retention time of 13.8 min.

### Cell viability assay

The anti-proliferative activity of citral toward AGS was evaluated using an MTT assay. The MTT assay was performed as those stated previously^33^. Cisplatin assisted as positive control and was likewise formulated. The DMSO solution considered as negative control.

### Colony formation

To determine the capability of proliferative activity and long-lasting survival aptitude of cancer cells colony formation assay was performed in a 6-well plate. The colony forming assay was performed as those stated previously^33^. Different concentration of citral was used to determine the colony formation of cell lines. Cell colony was stained by crystal violet, counted and graphically plotted. All statistical analysis were performed. 

### Wound healing Assay

The assay was performed according to the previous method^33^. The citral was used as dose-dependent manner and the cells were stained by crystal violet.

### Light microscopy

The density of 2 × 10^4^ cells per well were seeded on to 96-well culture plates and allowed to growfor 24 h. Then different concentration of citral were treated as previously described^33^. The morphological changes of cell images of both treated and untreated cells was captured a Leica DMIL LED equipped with an Integrated 5.0 Mega-Pixel MC 170 HD camera (Wetzlar, Germany).

### RNA isolation

AGS cell lines were cultured in 25 cm^2^ cell culture flasks and treated with different concentration of citral. After 48 h, the total RNA content was extracted by using RNeasy mini kit (Qiagen, Hilden, Germany). The isolated RNA was used for RNA sequencing.

### RNA sequencing and read mapping

In this study, the paired end (PE) RNAseq libraries of control and citral treated samples were prepared by using Truseq Stranded mRNA Prep Kit (Illumina) according to manufacture protocol. Then constructed RNAseq libraries were sequenced by Illumina Hiseq 2500 platform to obtain 101 base pair length reads. The generated each raw reads quality, removal of adaptor sequences, and low quality reads filtration was accessed through FASTX-Toolkit (http://hannonlab.cshl.edu/fastx_toolkit/). Further, obtained high quality reads were aligned with human genome (hg19) sequences using Tophat v2.0.13^34^ with default parameters and transcript expression was estimated with cuffdiff v.2.2.0^35^ using h19 genome annotations. The relative abundance of transcripts was normalized and measured in fragments per kb of exon per million fragments mapped (FPKM)^36^.

### Differential expressed genes and functional enrichment analysis

Cuffdiff from Cufflinks repository was used to identify differential expressed genes (DEGs) by comparing gene expression levels from control and citral treated groups. Further, Cuffdiff processed data was used to generate expression plots (Supplementary Fig. 2) using CummeRbund (http://compbio.mit.edu/cummeRbund/). The significant DEGs were selected with threshold of fold change more than 2 and P-value less than 0.05 by comparing control and citral treated groups. Initially, the up-regulated genes are selected for the functional enrichment analysis using the FunRich 3.1.3 tool. Where the distance between the nodes is set as 150 for producing the up-regulated gene network and up-regulated genes interacting with other relative genes (external genes).

Further, the gene list is considered to identify the significant Gene Ontology (GO) and to visualize the GO interaction network utilizing BiNGO 3.0.3 application in Cytoscape 3.6.1^37–39^. Further, functional enrichment analysis including GO and Kyoto Encyclopedia of Genes and Genomes (KEGG) pathway analysis was performed to the selected DEGs (fold change ≥ 2) using DAVID Bioinformatics Resources 6.8 to identify the apoptotic relative genes^40^. (https://david.ncifcrf.gov).

### Apoptotic cell propidium iodide staining

The propidium iodide staining assay was performed according to the previous method^33^. The appropriate number of AGS cell lines were seeded (5 × 10^4^/well) and treated with citral (10 and 20 μg/mL). The cells undergone apoptotic process were detected under fluorescence microscope using a Leica DMLB fluorescence microscope (Wetzlar, Germany).

### Hoechst staining

AGS cell lines (2 × 10^5^/well) were seeded and analyzing the apoptotic effect by citral in different concentration was measured by Hoechst staining 33342. The assay was performed according to the previous method^33^. The images were captured under fluorescence microscope using a Leica DMLB fluorescence microscope (Wetzlar, Germany).

### DNA fragmentation assay

The proper amount of cells were seeded and allowed for overnight to become well differentiated. The assay was performed according to the previous method^33^. After cell adhesion, citral was used at different concentration (5, 10 and 20 μg/mL) for the treatment. The gel was then examined under ultraviolet light and photographed.

## Supplementary information

Supplementary Info

Supplementary Info

Supplementary Info

Supplementary Info

Supplementary Info

Supplementary Info

Supplementary Info

Supplementary Info

## Data Availability

All the sequences were submitted to NCBI-SRA under accession number SRP150561.
