# Supplementary material for: Integrated transcriptome and in vitro analysis revealed anti-proliferative effect of citral in human stomach cancer through apoptosis
Source: Sci Rep. 2019 Mar 19;9:4883. doi: 10.1038/s41598-019-41406-8 (PMC6425008; doi:10.1038/s41598-019-41406-8)
Supplement: Supplementary file 1 — Supplementary Info [file 41598_2019_41406_MOESM1_ESM.pdf]

Research Article

Integrated transcriptome and *in vitro* analysis revealed anti-proliferative effect of citral in human stomach cancer through apoptosis

Sri Renukadevi Balusamy[a†*], Sivasubramanian Ramani[a], Sathishkumar Natarajan[b], Yeon Ju Kim[c*], Haribalan Perumalsamy[c†*]

[a]*Department of Food Science and Biotechnology Sejong University, Gwangjin-gu, Seoul, Republic of Korea*

[b]*Department of Horticulture, Sunchon National University, Suncheon, Republic of Korea*

[c]*Graduate School of Biotechnology, College of Life Science, Kyung Hee University, Yongin, 446-701, Republic of Korea*

Running Head: RNA- seq and *in vitro* study of citral induced apoptosis

[†]These authors contributed equally for this manuscript

Corresponding authors

Prof. Sri Renukadevi Balusamy, Department of Food Science and Biotechnology Sejong University, Gwangjin-gu, Seoul, 04489, Republic of Korea. +82-2-6935-2611, srirenukadevibalusamy@gmail.com

Dr. Haribalan Perumalsamy, Graduate School of Biotechnology, College of Life Science, Kyung Hee University, Yongin, 446- 701, Republic of Korea. +82-10-2902-4695, harijai2004@gmail.com

Prof. Yeon Ju Kim, Graduate School of Biotechnology, College of Life Science, Kyung Hee University, Yongin, 446- 701, Republic of Korea. +82-10-2902-4695, yeonjukim@khu.ac.kr

Supplementary Figures Legends

Supplementary Fig. 1 NMR spectrum of citral. (A) ^{1}HNMR (B) ^{13}CNMR spectra

Supplementary Fig. 2. RNA isolation quality and Phred score of control and citral treated samples in AGS. (A) RNA quality of control sample (B) RNA quality of citral treated sample (C) Phred score of control sample from RNA-seq (D) phred score of citral treated sample from RNA seq.

Supplementary Fig. 3. Density distribution plot of significantly expressed genes from transcriptome libraries of AGS. (A) The expression level was measured based on log 10 value of fragments per kb of exon per million fragments mapped (FPKM) (B) Volcano plot of significantly expressed genes.

Supplementary files

Supplementary file S1. Interacting external relative genes with upregulated DEG

Supplementary file S2. Interacting partner genes between DEG

Supplementary file S3. Biological Process identified based on gene enrichment analysis

Supplementary file S4. Molecular function identified based on gene enrichment analysis

Supplementary file S5. Cellular components involved based on gene enrichment analysis

Supplementary file S6. The list of DEG in citral treatment

Supplementary file S7. Detailed information about the KEEG pathways of DEG

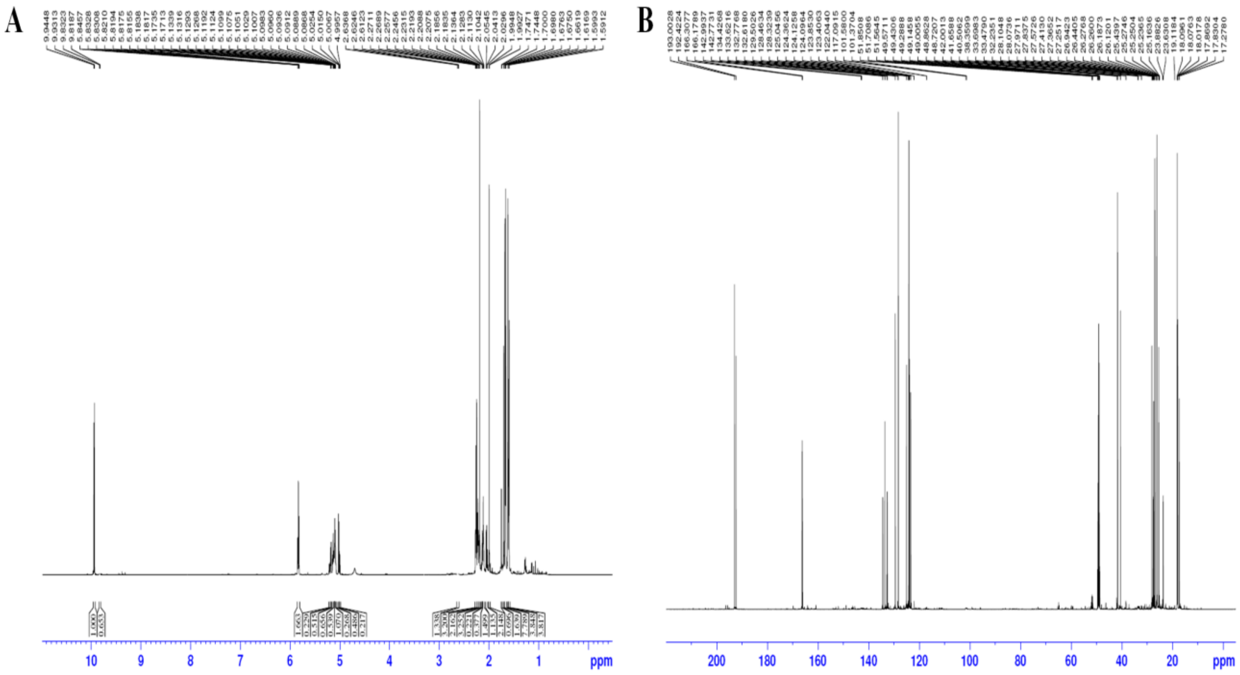

Supplementary Fig. 1

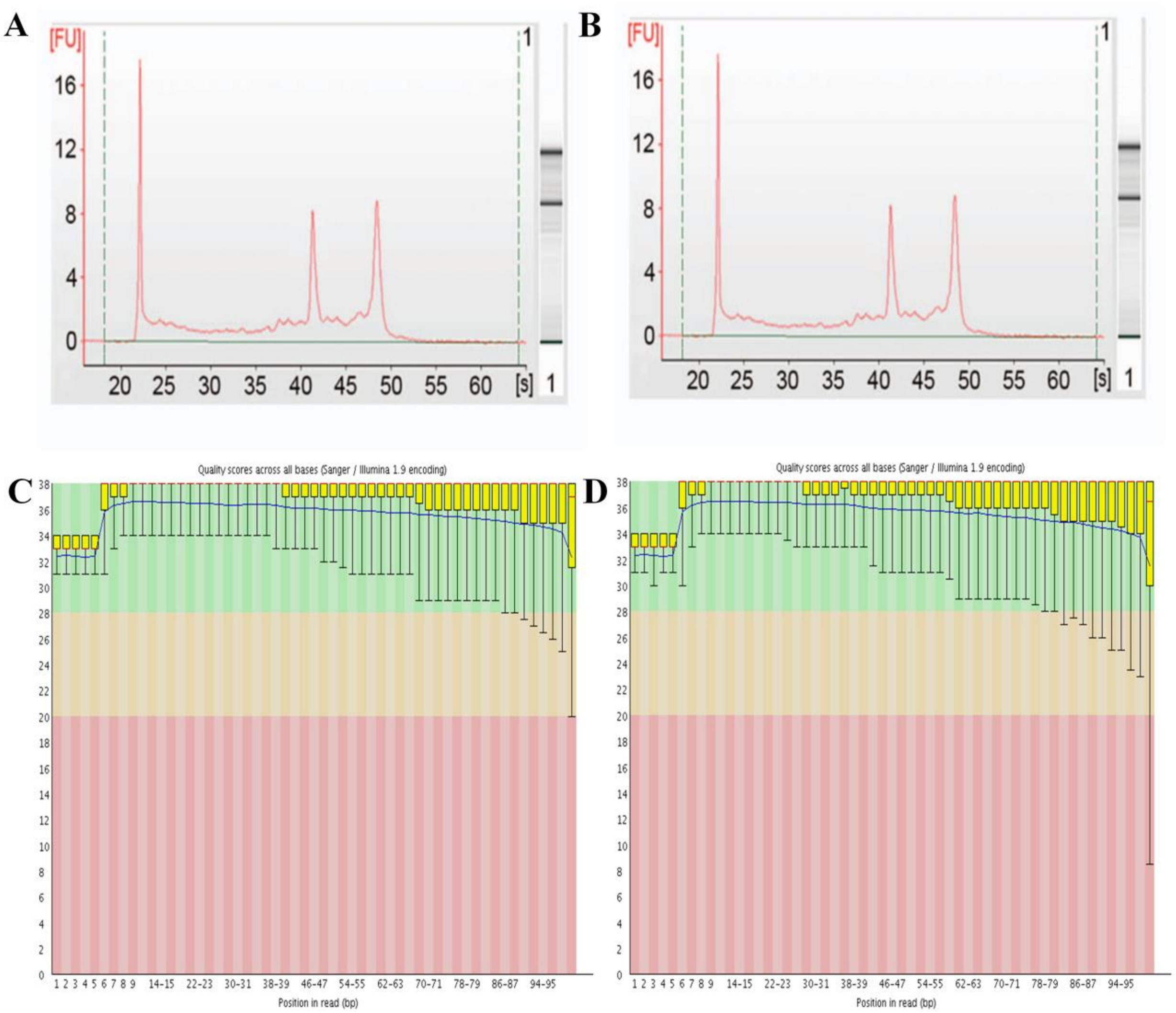

Supplementary Fig. 2

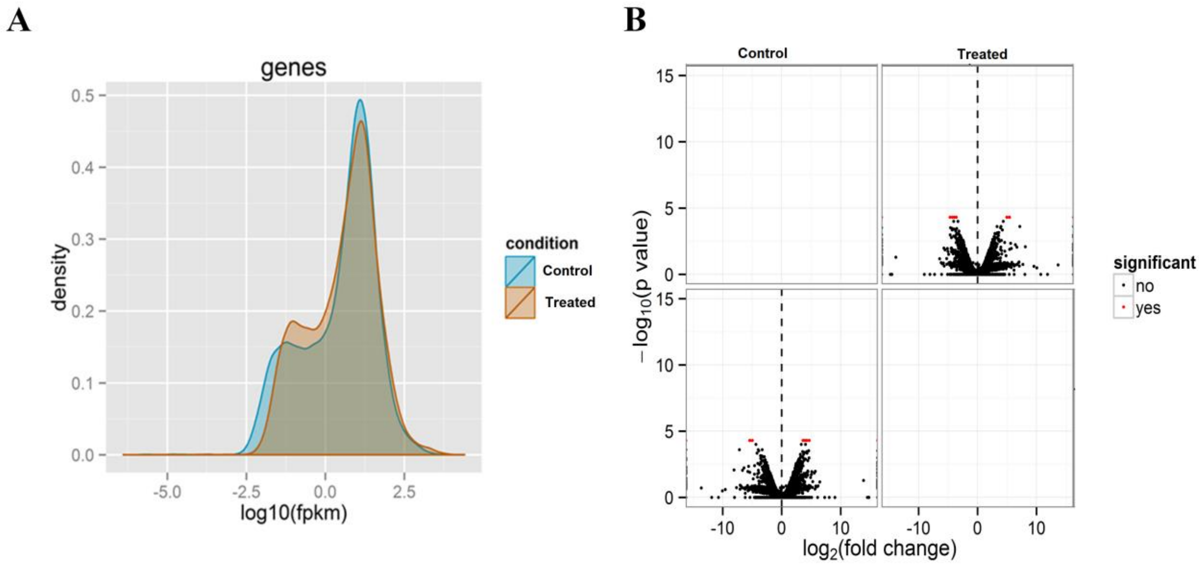

Supplementary Fig. 3
